# Ultrasonic manifestations of diffuse pericardial mesothelioma masquerading as pericardial effusion: a case report

**DOI:** 10.3389/fonc.2026.1921935

**Published:** 2026-10-06

**Authors:** Shuxu Zhu, Yibo Zhou

**Affiliations:** Department of Ultrasound, Affiliated Jinhua Hospital, Zhejiang University School of Medicine, Jinhua, Zhejiang, China

**Keywords:** case report, diagnosis, pericardial effusion, primary pericardial mesothelioma, ultrasonic features

## Abstract

**Background:**

Primary pericardial mesothelioma (PPM) is an exceedingly rare, highly invasive cardiac neoplasm with insidious onset and non-specific clinical symptoms. Due to the lack of typical early manifestations and effective routine diagnostic methods, PPM is often misdiagnosed and missed, resulting in delayed treatment and poor prognosis.

**Case presentation:**

A 42-year-old male presented with 6 months of recurrent chest tightness and pain, worsening over 2 months, and two prior admissions for unexplained pericardial effusion. On admission, he had tachycardia, distant heart sounds, bilateral lower extremity edema, and a 3×2 cm hard, fixed left supraclavicular lymph node. Imaging confirmed pericardial effusion with abnormal pericardial lesions. Ultrasound-guided pericardiocentesis yielded only a small amount of red jelly-like material, with cytology suggesting epithelioid neoplastic cells. Supraclavicular lymph node biopsy confirmed metastatic malignant mesothelioma. Nivolumab immunotherapy showed no improvement. Pericardial tumor resection revealed diffuse, fish-flesh-like tumor invading the myocardium and great vessels. Final pathology confirmed epithelioid-type PPM.

**Conclusion:**

Patients with recurrent unexplained pericardial effusion and atypical sonographic features, such as flocculent echoes and irregular pericardial thickening, should raise suspicion for PPM. Early recognition of these signs and timely pathological confirmation are critical to avoid diagnostic delays and may improve outcomes.

## Introduction

Primary pericardial mesothelioma(PPM) is an extremely rare primary pericardial tumor. It is difficult to achieve an accurate diagnosis at the early stage, and often misdiagnosed as other pericardial diseases (1). We present a case of pericardial mesothelioma, detailing the patient’s clinical course from symptom onset to surgical treatment. We also analyze its echocardiographic features and review the relevant literature.

## Case presentation

A 42-year-old male presented to our hospital with a 6-month history of recurrent chest tightness and pain, with progressive worsening over the prior two months. He had been hospitalized twice previously for pericardial effusion. The patient had no family history of pericardial mesothelioma or inherited thoracic neoplasms and denied prior asbestos exposure. His blood pressure was 115/89 mmHg. Physical examination revealed a firm, poorly mobile left supraclavicular lymph node measuring 3 × 2 cm. Cardiac auscultation showed distant heart sounds, and bilateral lower limb edema was noted. Initial admission blood tests showed elevated BNP, CYFRA 21–1 and inflammatory markers (**Table 1**).

**Table 1:** Serial laboratory parameters of the patient.

Laboratory index	Admission	Pre-operation	Post-operation	Reference range
BNP	2000 pg/mL	2140 pg/mL	3450 pg/mL	0–125 pg/mL
hs-CRP	8.23 mg/L	25.39 mg/L	11.30 mg/L	<8.0 mg/L
CYFRA 21-1	55.3 ng/mL	153.6 ng/mL	NA	<3.3 ng/mL
NSE	NA	76.8 ng/mL	NA	<20 ng/mL
WBC count	12.91 ×10^9/L	13.79 ×10^9/L	20.17 ×10^9/L	3.50–9.50 ×10^9/L
Hemoglobin	146 g/L	172 g/L	130 g/L	130–175 g/L
Platelet count	507 ×10^9/L	445 ×10^9/L	453 ×10^9/L	125–350 ×10^9/L
LDH	192 U/L	342 U/L	198 U/L	120–246 U/L

BNP, B-type natriuretic peptide; hs-CRP, high-sensitivity C-reactive protein; CYFRA 21-1, cytokeratin fragment antigen 21-1; NSE, neuron-specific enolase; WBC, white blood cell; LDH, lactate dehydrogenase.

Transthoracic echocardiography demonstrated anechoic areas in the pericardium, accompanied by flocculent hyperechoic specks (**Figure 1**). Chest computed tomography (CT) revealed low-density lesions within the pericardial cavity (**Figure 2**). At initial presentation, no discrete solid pericardial mass was identified on imaging. Given the patient’s young age, absence of asbestos exposure, and elevated inflammatory markers, recurrent pericardial effusion was most suggestive of idiopathic inflammatory pericarditis. PPM is exceedingly rare, and therefore was not the leading diagnostic consideration. Then we conducted ultrasound-guided pericardiocentesis to drain pericardial effusion. The procedure was conducted following standard protocols. Needle insertion was uncomplicated, yet nearly no fluid was aspirated. Only a small quantity of red gelatinous substance was collected for smear cytology, which identified epithelioid neoplastic cells.

**Figure 1:**
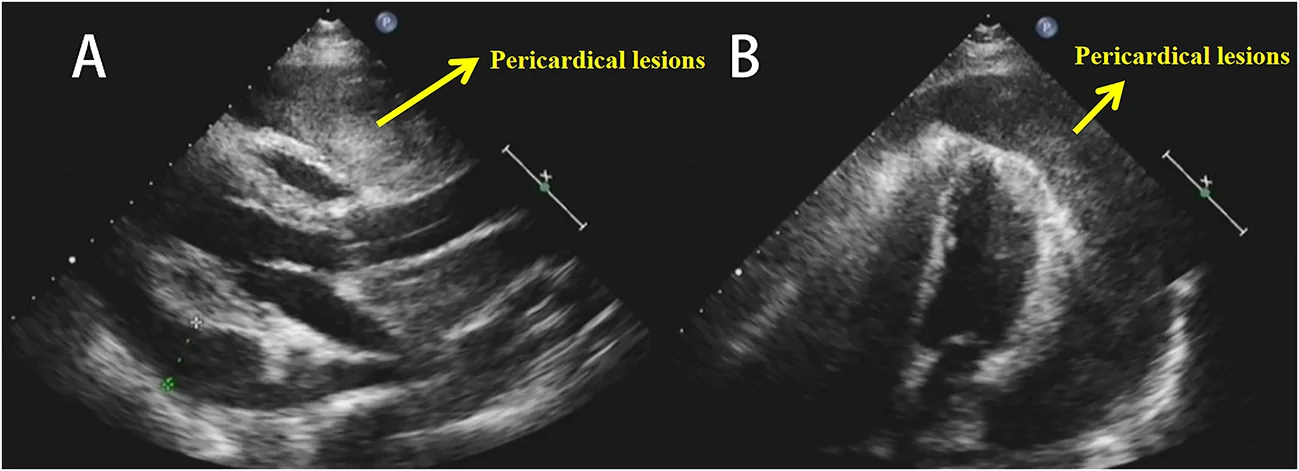
**(A)** Parasternal long-axis view showed pericardial anechoic areas with flocculent hyperechoic specks; the pericardial lesion adjacent to the anterior wall of the right ventricle was 29 mm thick, and the lesion beside the left ventricular posterior wall measured 25 mm. **(B)** Apical four-chamber view revealed pericardial anechoic areas with flocculent hyperechoic specks, with a lesion thickness of 21 mm at the cardiac apex.

**Figure 2:**
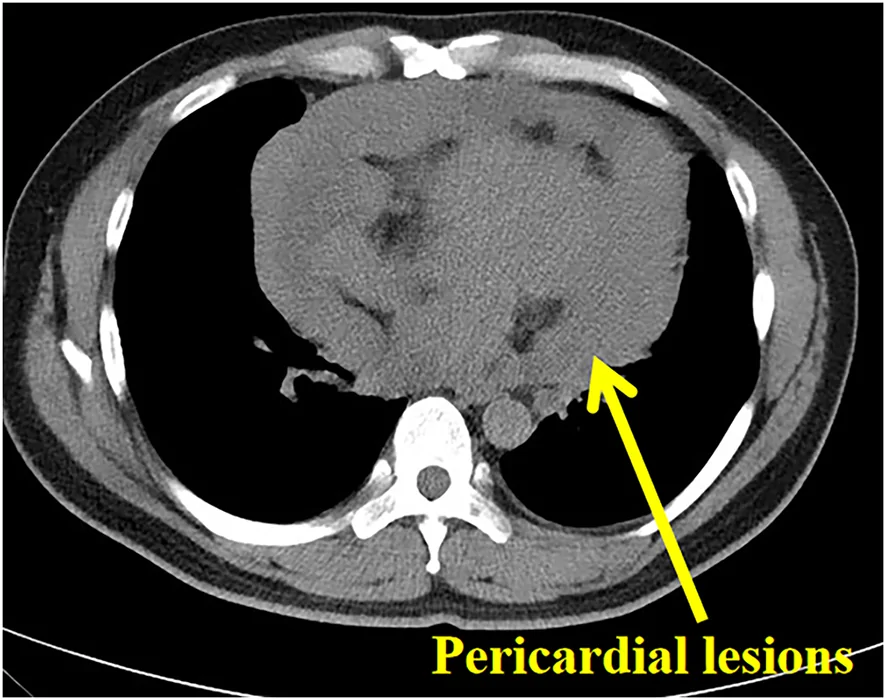
Chest CT demonstrated a low-attenuation lesion encasing the heart.

To clarify the pathological diagnosis, we performed ultrasound-guided core needle biopsy of the left supraclavicular lymph node, given the patient’s poor physical condition and refusal of large-bore needle pericardial biopsy. Pathological examination confirmed lymph node metastasis of malignant mesothelioma. After definite diagnosis, the patient was initiated on nivolumab immunotherapy at a dose of 200mg administered intravenously every 2 weeks. Given that advanced malignant pericardial mesothelioma lacks standardized first-line therapeutic regimens, nivolumab was used as an off-label therapeutic attempt. Ten days after the first cycle, the patient experienced progressive worsening of chest distress and dyspnea. In view of the rapidly deteriorating clinical status, surgical tumor resection was performed for palliative decompression.

Intraoperative findings revealed diffuse tumor involvement of the entire pericardium. The tumor exhibited a fish-flesh-like gross appearance and infiltrated the myocardium and surrounding great vessels. Combined with pre-operatively confirmed supraclavicular lymph-node metastasis, these findings indicated far-advanced disease. Concurrent local tissue invasion and regional lymph-node spread rendered complete curative resection technically unfeasible, which partly accounts for the poor prognosis of this malignancy. Postoperative pathological examination verified the final diagnosis of epithelioid malignant pericardial mesothelioma (**Figure 3**). Immunohistopathological results showed the following immunoreactivity profile: CK5/6(+), calretinin(+), D2-40(+), mesothelin(+), CK7(+), CK-P(+), p53(+), ERG(+), ALK(–), CK20(–), TTF-1(–), SALL4(–), SOX10(–), desmin(–), and Ki-67 (60% positive). However, on the third postoperative day, the patient developed sudden heart failure and subsequently died.

**Figure 3:**
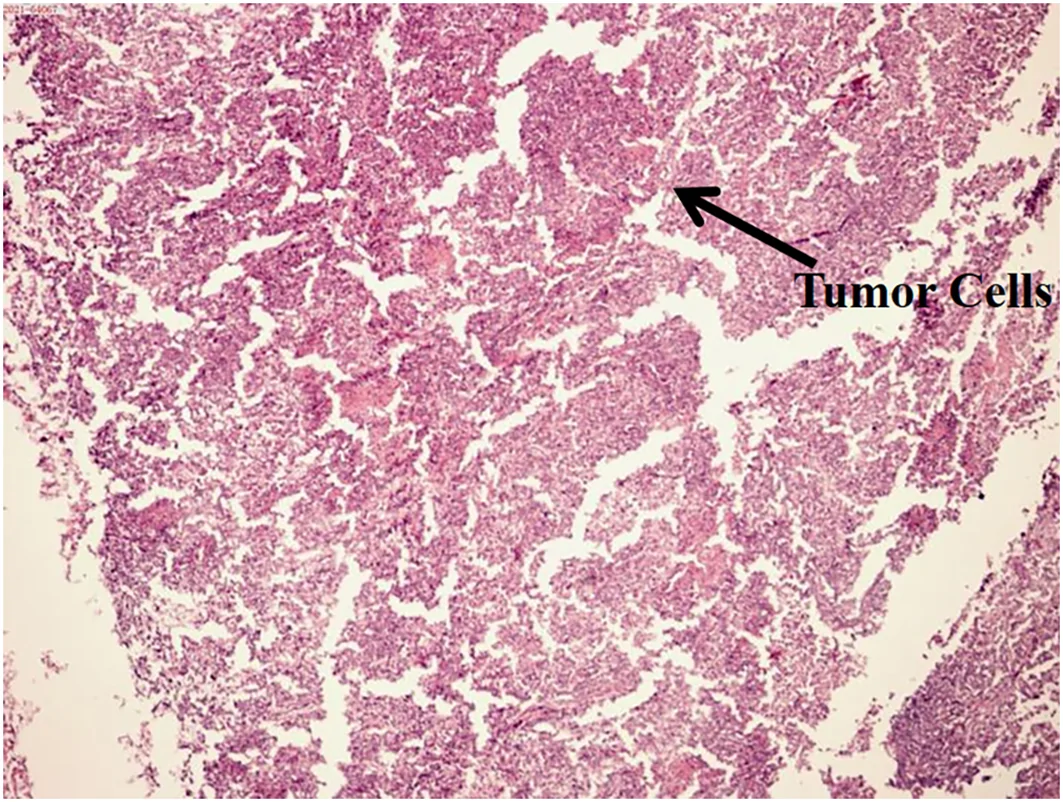
Histological examination, hematoxylin-eosin (HE) staining, magnification ×100. Tumor cells were arranged in solid sheets or nests. The lesions consisted of epithelioid cells with round or polygonal morphology. Mild cellular atypia was noted, and the cytoplasm was eosinophilic.

**Table 1** summarizes serial laboratory examinations obtained at admission, pre-operation and post-resection stages. **Table 2** presents a chronological clinical timeline to fully demonstrate the patient’s diagnostic procedures and treatment course as requested in the review comments.

**Table 2:** Clinical timeline of diagnosis and treatment.

Time point	Key clinical events	Diagnostic examinations	Interventions & outcomes
6 months prior to admission	Onset of recurrent chest tightness and pain; symptoms progressively aggravated over the prior 2 months	NA	Hospitalized twice for pericardial effusion
Admission (Day 0)	Admission to our hospital	Physical examination: firm left supraclavicular lymph node (3×2 cm), distant heart sounds, bilateral lower limb edema Transthoracic echocardiography: pericardial anechoic areas with flocculent hyperechoic specks CT: low-density lesions within the pericardial cavity	Initially considered as idiopathic simple pericardial effusion
Pericardiocentesis (Day 4)	Persistent pericardial symptoms; initial diagnostic workup	Ultrasound-guided pericardiocentesis to drain pericardial effusion	Pericardiocentesis obtained only small red gelatinous substance; smear cytology revealed epithelioid neoplastic cells.
After pericardiocentesis (Day 6)	Definitive pathological diagnosis required	Ultrasound-guided core needle biopsy of left supraclavicular lymph node	Pathology: lymph node metastasis of malignant mesothelioma
After confirmed diagnosis (Day 11)	Initiation of antitumor therapy	NA	Nivolumab immunotherapy initiated; No symptomatic relief was observed after 10 days of nivolumab treatment
Operation day (Day 21)	Surgical intervention	NA	Surgical resection of pericardial tumor performed
Postoperative (Day 24)	Sudden clinical deterioration	Diagnosed with acute heart failure	Patient passed away due to progressive acute heart failure

## Discussion

PPM is an extremely rare clinical malignancy, accounting for less than 1% of all mesothelioma cases, with only over 100 cases reported worldwide to date (2, 3).This disease follows an insidious clinical course and is often asymptomatic in its early phase, and is associated with a poor overall prognosis (4). Initial manifestations are predominantly chest tightness and chest pain, often accompanied by pericardial effusion—findings that closely overlap with those of tuberculous and idiopathic pericarditis, leading to a high rate of misdiagnosis and missed diagnosis (5).

Notably, this patient was hospitalized twice due to pericardial effusion, yet the underlying cause was not fully identified. The disease was eventually diagnosed at an advanced stage with lymph node metastasis and extensive pericardial infiltration, offering an important clinical reminder for clinicians.

To further summarize the clinical, imaging, therapeutic and prognostic features of primary pericardial mesothelioma (PPM) and compare with the present case, previously reported PPM cases in recent literature were reviewed and summarized in **Table 3**.

**Table 3:** Comparative analysis of published primary pericardial mesothelioma cases.

Reference	Age	Sex	Main clinical manifestations	Imaging features	Treatment strategy	Clinical outcome
(2)	46	Female	Fever and chest pain	PET-CT: severe pericardial effusion and hypermetabolic pericardial thickening	Dual Immunotherapy: nivolumab and ipilimumab)	Alive, partial/complete metabolic remission
(4)	70	Male	Chest tightness	CT and ultrasound: Massive recurrent pericardial effusion PET-CT: pericardial _18_F-FDG nodule	Pericardial drainage every 3 months	Alive, stable disease for 3 years without progression
(7)	78	Male	Transient loss of consciousness and falls	CT and ultrasound: cardiac enlargement and pericardial effusion	Conservative follow-up	Died of pneumonia
(8)	60	Male	Progressive dyspnea	MRI and ultrasound: Diffuse whole pericardial thickening, periaortic involvement	Pericardiectomy	Died of heart failure
(9)	35	Female	Shortness of breath	CT: pericardia effusion and pericardial thickening	Pericardiectomy	Recovered postoperatively
(12)	82	Male	Dyspnea, lower leg edema	CT and ultrasound: massive left pleural effusion, pericardia effusion and pericardial thickening	Pericardiectomy; carboplatin and pemetrexed adjuvant chemo	Smooth postoperative recovery
(15)	65	Female	Dyspnea and palpitations	Ultrasound: pericardial effusion and hyper-enhanced pericardial mass	Palliative pericardiotomy	Died 2 years after diagnosis
(20)	74	Male	Fever and mildly tachycardic	CT and PET-CT: large pericardial mass, moderate pericardial effusion, lymphadenopathy	Pericardiectomy; nivolumab and ipilimumab immunotherapy	Stable condition
(21)	68	Male	Dyspnea on exertion, bilateral lower-extremity edema	CT and ultrasound: pericardial effusion, posterior pericardial mass	Surgical resection; cisplatin and pemetrexed adjuvant chemo	Died of local recurrence 27 months after surgery

Pathological examination remains the gold standard for the diagnosis of PPM. However, pericardial puncture and biopsy are technically challenging and poorly tolerated by most patients, making imaging examinations the primary approach for early clinical diagnosis of this rare disease.

As a non-invasive and radiation-free imaging modality, echocardiography serves as the first-line diagnostic tool for the evaluation of pericardial diseases. Compared with CT, it enables real-time assessment of the characteristics of pericardial effusion and continuous monitoring of cardiac hemodynamic changes. Echocardiography can also clearly visualize pericardial masses and myocardial motion, while allowing measurement of pericardial thickness, thereby playing a critical role in the diagnosis and clinical evaluation of various pericardial disorders (6). Typical echocardiographic manifestations in patients with primary pericardial malignancies mainly include pericardial effusion, pericardial space-occupying lesions, pericardial thickening (7, 8).

Literature reviews show that the majority of patients with PPM present with pericardial effusion (9). Such effusion is often hemorrhagic and accumulates rapidly. On ultrasound, such effusion may show poor sound transmission with punctate or flocculent internal echoes (10, 11), an imaging finding that can be easily confused with diffuse infiltrative tumor. In our case, the absence of this typical effusion and presence of jelly-like material highlight the diagnostic challenge of this condition. Additionally, large-volume pericardial effusion can lead to secondary cardiac tamponade, a common complication in patients with PPM (12).

In echocardiography, some patients with PPM present with extensive, heterogeneous, solid mass-like echoes within the pericardial cavity, which may involve any segment of the pericardium (13). In clinical differential diagnosis, benign pericardial tumors and metastatic lesions must be carefully excluded. PPM often causes diffuse thickening of the pericardium, with typical sonographic features including irregular thickening of the pericardial wall and heterogeneous infiltrative echoes within the pericardial space. Some cases have been reported to manifest clinical features mimicking constrictive pericarditis (14).

When conventional echocardiography fails to clarify the nature of a pericardial mass, contrast-enhanced echocardiography (CEE) serves as a safe and simple alternative. It can assess the vascularity of the lesion, offering a diagnostic option for patients who refuse pericardiocentesis or are too critically ill to undergo invasive procedures. In a study by Wang et al. (15), contrast-enhanced echocardiography was applied to PPM patients presenting with pericardial masses on two-dimensional ultrasound. The authors observed multiple vessels extending from the adjacent myocardium into the mass, resulting in marked enhancement that exceeded that of the surrounding myocardial tissue. In contrast, benign pericardial tumors typically showed no enhancement or only mild enhancement.

Clinical features that warrant early consideration of PPM include refractory recurrent pericardial effusion, gelatinous pericardial aspirate, supraclavicular lymphadenopathy even in the absence of a discrete solid mass. Differential diagnoses for PPM include the following conditions: ① Pericardial metastatic tumors: Echocardiographic features are non-specific, often showing nodules of varying sizes within the pericardium associated with variable degrees of pericardial effusion. Patients usually have a known primary malignancy, most commonly lung or breast cancer, and a detailed clinical history is essential for differentiation (16). ② Benign pericardial tumors: These include teratomas, lipomas, and pericardial cysts. On ultrasound, the masses typically have well-defined borders, while pericardial effusion and diffuse pericardial thickening are rare, and cardiac tamponade is uncommon (17). MRI with fat-saturation sequences can help distinguish teratomas and lipomas from other lesions. ③ Tuberculous pericarditis: It presents with diffuse pericardial thickening, adhesions, and calcification, without evidence of pericardial masses. In contrast to PPM, pericardial effusion develops more gradually (18).

Based on published literature, the overall prognosis of patients with PPM is extremely poor, with a median survival time ranging from 3 to 10 months (14). PPM is generally insensitive to both radiotherapy and conventional chemotherapy, while immunotherapy has shown limited but promising efficacy in some cases (19, 20). Surgical resection is considered one of the main treatment modalities; however, most patients only undergo palliative procedures to relieve symptoms, and curative resection is rarely achieved (21).

This report improves clinical understanding of rare primary pericardial mesothelioma. As a single-case study, it has inherent limitations, and conclusions cannot be generalized to broader patient populations. Clinical observation was limited by the early death of the patient after surgical intervention.

## Conclusions

PPM is an extremely rare malignancy with an insidious onset and non-specific clinical manifestations, which frequently leads to misdiagnosis and delayed diagnosis. Patients with recurrent unexplained pericardial effusion combined with atypical ultrasonic features, including irregular pericardial thickening or intracavitary echo abnormalities such as flocculent echoes, should arouse early clinical suspicion of primary malignant pericardial mesothelioma. Careful evaluation of these characteristic signs is critical for identifying potential neoplastic pericardial lesions.

## Data Availability

The original contributions presented in the study are included in the article/supplementary material. Further inquiries can be directed to the corresponding author.
